# Correction to: Sometimes noise is beneficial: stream noise informs vocal communication in the little torrent frog *Amolops torrentis*

**DOI:** 10.1007/s10164-017-0537-5

**Published:** 2017-12-01

**Authors:** Longhui Zhao, Bicheng Zhu, Jichao Wang, Steven E. Brauth, Yezhong Tang, Jianguo Cui

**Affiliations:** 1 Chengdu Institute of Biology, Chinese Academy of Sciences, Chengdu, 610041 Sichuan China; 2 University of Chinese Academy of Sciences, Beijing, 100049 China; 3 Ministry of Education Key Laboratory for Tropical Plant and Animal Ecology, College of Life Sciences, Hainan Normal University, Haikou, 571158 Hainan China; 4 Department of Psychology, University of Maryland, College Park, MD 20742 USA

## Correction to: J Ethol (2017) 35:259–267 https://doi.org/10.1007/s10164-017-0515-y

The article, “Sometimes noise is beneficial: stream noise informs vocal communication in the little torrent frog Amolops torrentis”, written by Longhui Zhao, Bicheng Zhu, Jichao Wang, Steven E. Brauth, Yezhong Tang, and Jianguo Cui, was originally published Online First without open access. After publication in volume 35, issue 3, pages 259–267 the author decided to opt for Open Choice and to make the article an open access publication. Therefore, the copyright of the article has been changed to © The Author(s) 2018 and the article is forthwith distributed under the terms of the Creative Commons Attribution 4.0 International License (http://creativecommons.org/licenses/by/4.0/), which permits use, duplication, adaptation, distribution and reproduction in any medium or format, as long as you give appropriate credit to the original author(s) and the source, provide a link to the Creative Commons license, and indicate if changes were made.

